# Abdominal Section in a Case of Cyst of the Mesentery, with Remarks

**Published:** 1892-02

**Authors:** J. Adrian Goggans

**Affiliations:** Alexander City, Ala., Member of the Board of Medical Examiners of Tallapoosa County; Senior Counsellor of the Medical Association of the State of Alabama; Fellow of the Southern Surgical and Gynæcological Society ; Fellow of the British Gynæcological Society


VOL. VIII. FEBRUARY, 1892. NO. 12.

TO CONTRIBUTORS.

Articles for publication must reach this office not later than 15th inst.; and are expected to be published exclusively in this JOURNAL. Extra copies of JOURNAL furnished contributors when requested. Reprints by special arrangement. Publication of an article does not necessarily imply endorsement of the views therein expressed.

ATLANTA MEDICAL AND SURGICAL JOURNAL, Post-office box 431.

### Original Communications.

### ABDOMINAL SECTION IN A CASE OF CYST OF THE MESENTERY, WITH REMARKS.*

BY J. ADRIAN GOGGANS, M. D., ALEXANDER CITY, ALA.,
Member of the Board of Medical Examiners of Tallapoosa County; Senior Counsellor of the Medical Association of the State of Alabama; Fellow of the Southern Surgical and Gynæcological Society; Fellow of the British Gynæcological Society.

I have been induced to write this paper from the fact that cysts of the mesentery are extremely rare, and operations for their removal have proven to be most generally fatal. Delagenière[1] presented specimens of a tumor of the mesentery to the Anatomical Society of Paris, removed from a patient by Guyon. The patient was a woman forty-five years of age, suffering from epilepsy. The tumor occupied the left hypochondriac and

*Read before the Southern Surgical and Gynæcological Society in Richmond, Va., November 12, 1891.

[1] J. Ewing Mears: Sajous' Annual of Universal Medical Science, Vol. iii.

lumbar regions, and was as large as a fœtal head. It was increasing in size, and caused such abdominal pains that Guyon decided to remove the fluid by aspiration. He removed thirty-two ounces of a brownish fluid which contained albumin, urea, phosphates and chlorides. Within two weeks after the aspiration the tumor had increased to the size it had attained before the operation, and the patient suffered such pains that Guyon decided to open the abdomen and attempt to remove the cyst. After opening the abdomen, he removed by aspiration twenty-eight ounces of fluid, and upon examination the cyst seemed to originate in the meso-colon. It was finally enucleated, after having ligated many large blood-vessels. The patient died on the seventh day after the operation. Sir Spencer Wells[2] has performed two operations for tumors of the mesentery ; one of them was solid, and the other was cystic. He incised and drained the cyst, but the patient died within a few weeks after the operation. The solid tumor was successfully removed by enucleation. Greig-Smith[3] mentions seven cases of lipoma of the mesentery, in which operations were performed for their removal. He describes them as follows: Terrillon[4] presented a patient to the Academy of Medicine of Paris, from whom he had removed lipoma of the mesentery by enucleation. Homans[5] has performed two operations for the removal of lipoma of the mesentery. One of them was a male, thirty-nine years of age. His first attempt to remove the tumor in this case was a failure, but a successful operation was performed some months afterward. The patient finally died from shock. The second case was that of a woman sixty years of age, in every way similar to the first case. This patient likewise died from shock. Dr. Calvin Ellis, according to Homans, had a similar case. Three cases of lipoma of the mesentery are described in the *Transactions of the Pathological Society of London.* And Cooper Forster[6], in

---

[2] Lancet, May 5, 1883.

[3] Greig Smith : Abdominal Surgery.

[4] Journal of the American Medical Association.

[5] Lancet, 1883, p. 449.

[6] Greig-Smith : Abdominal Surgery.

1868, showed a large fatty tumor at the Pathological Society of London, removed from a woman after death. Pèan[7] describes three operations for cystic tumors of the mesentery, with only one success. Watts[8] describes another operation for cyst of the mesentery. In this case the tumor was mistaken for an ovarian cyst. Carter[9] likewise described an operation for the removal of a cyst of the mesentery in a female patient forty-four years of age. It was of two years' growth, and contained sixteen pints of fluid. The attachments of this cyst to the spine and lumbar region were so firm, and it was so closely encircled by coils of the small intestine, that the attempt at its removal was abandoned. The operator then incised and drained the cyst, and stitched the incised lips of the cyst to the abdominal walls. The patient finally died from septicæmia and hemorrhage. Greig-Smith[10] says that he knows of two cases of cyst of the mesentery in which operations were performed for their removal by his friends, but that they have not yet been published.

Dr. Granville Bantock describes a case of cyst of the mesentery in which the tumor was about the size of a Mandarin orange. It was in a young girl fifteen years of age She sometimes had a temperature of 105° F. Sir Joseph Lister agreed with Dr. Bantock as to the necessity of its removal, and, consequently, the operation was performed on February 16, 1886. An incision was made in the abdomen, along the outer border of the rectus muscle. The tumor was covered by the mesentery, whose blood vessels were enormously enlarged, and it lay in close proximity to the left kidney, for which it was mistaken. Many ligatures were required, a drainage-tube was inserted, and the patient made a tedious recovery. The tumor weighed one pound, and is now in the Museum of the Royal College of Surgeons. Mr. Eve made the following report on this tumor : "I am quite at a loss to explain its origin. The tumor

---

[7] Tumeurs de l'Abdomen.

[8] American Journal of Obstetrics, 1879.

[9] British Medical Journal, January 6, 1883.

[10] Personal communication.

[11] Personal communication and Lancet, 1887.

is composed of a number of intercommunicating loculi, separated by numerous septa, and containing a glairy fluid. The situation being so unusual, I conjecture that it originated from some fœtal structure—possibly some of the rudiments of the permanent kidney. It was too high up for the Wolffian body. The other possibilities that occur to me are, that it was an ovarian cyst which had become separated by twisting of pedicle, or that it was of the same nature of cysts that appear in the great omentum." Dr. Bantock says: "I take Mr. Eve's first explanation as being the probable correct explanation of the origin of this tumor. It was situated too much behind the mesentery for an ovarian tumor which had been separated from its attachments."

The patient upon whom I operated for a mesenteric cyst was Miss S. L. M., aged twenty-one years, daughter of a physician of Columbus, Ga., fair complexion, and weight, when in health, one hundred and twenty pounds. Family history good. I saw her for the first time on February 7, 1891. She stated to me that she had been out of health for about two years, but referred the beginning of her abdominal trouble to a cold which she contracted while in New Orleans about May, 1890. It was not until November or December, 1890, that she called her father's attention to the fact that her abdomen was becoming larger. She had been seen by several physicians, and treated for abdominal dropsy. At the time of my first visit, her abdomen was quite large, her pulse 110 per minute, and temperature 100° F. I made the diagnosis of an abdominal cyst, the tumor occupying mostly the left side of the abdomen, extending from under the ribs into the left lumbar region, dipping down into the pelvis, and extending three inches to the right side of the median line of the abdomen into the right side. I decided to remove some of the fluid by aspiration for examination, and did remove about one quart. It was a thin, dark-colored fluid, and contained albumin phosphates and chlorides. I saw her again on February 13, 1891. She had suffered much pain, accompanied by nausea and vomiting, and was more prostrated than before the operation. An operation for the removal of the tumor was proposed, and she readily consented to have it performed, February 24, 1891, being the day selected for the operation. In the meantime I asked my friend, Dr. W. E. B. Davis, of Birmingham, Ala., to see her with me, and assist me with the operation. Consequently, he saw her on February 24th. Upon examination he concurred in the opinion that an abdominal cyst existed, but we were unable to explain its origin and attachments.

The operation was performed with the assistance of Dr. Davis,[1] Dr. J. W. Mitchell, of Hamilton, Ga., and Dr. T. S. Mitchell and Dr. Phillips, of Columbus, Ga. The strictest antiseptic measures were carried out. The abdominal incision was made in the linea alba, extending two inches above and two inches below the umbilicus. On introducing the hand, it was evident that the attachments of the cyst were high up in the abdomen and in the region of the left kidney. The left kidney, however, was easily recognized. The walls of the cyst were covered with omentum. Many large blood vessels and several loops of small intestine were encircling it, being deeply imbedded in the walls of the cyst. An attempt to enucleate the cyst was followed by such profuse hemorrhage that the idea of enucleation was abandoned. Consequently, a point on the walls of the cyst, as remote as possible from blood vessels and loops of intestines, was selected for incision and drainage; more than a gallon of dark, thin-colored fluid was evacuated. The cyst was then irrigated with hot water, its incised lips stitched to the upper angle of the abdominal incision, an ordinary six-inch glass drainage-tube introduced to the bottom of the sac, and the abdominal incision closed with silk-worm-gut sutures. The general direction of this tube was upward and backward. From the appearance of the sac and its deep attachments, both Dr. Davis and myself came to the conclusion that it was retro-peritoneal. In other words, that the cyst was between the layers of the mesentery. The time consumed in performing the operation was about twenty-five minutes. On the morning of the operation the

[1] I wish to express my sincere appreciation of the skill and invaluable assistance rendered me by Dr. Davis during this operation.

patient's pulse was 120 per minute, and temperature 100° F. She came out from the anæsthetic well, and at 10 P. M. of the same day her temperature was 100⅗° F., and pulse 130 per minute.

*February* 25. 7 A. M., temperature 99°, pulse 104. 6 P. M., temperature 99⅕°, pulse 110.

26*th*. 7 A. M., temperature 98½°, pulse 110. 6 P. M., temperature 100⅕°, pulse 106.

The fluid has been drawn from the sac at intervals of three or four hours, and the sac thoroughly irrigated with warm water. Gave an enema of warm water, and the bowels have moved well.

27*th*. 7 A. M., temperature 99½°, pulse 104. 10 P. M., temperature 99½°, pulse 110.

28*th*. 7 A. M., temperature 100⅕°, pulse 112.

*March* 1. 7 A. M., temperature 98⅘°, pulse 96.

Has had much nausea and vomiting from time of operation until March 1st, much of which I attribute to the intimate connection between the walls of the cyst and the small intestines. She is now taking a little food. The sac is being irrigated daily, and the drainage-tube being gradually withdrawn.

2*d*. 7 A. M., temperature 98⅘°, pulse 100.

3*d*. 7 A. M., temperature 98⅘°, pulse 94. 8 P. M., temperature 98⅕°, pulse 96.

5*th*. 7 A. M., temperature 99°, pulse 96. 6 P. M., temperature 99⅖°, pulse 100.

8*th*. 6 A. M., temperature 98½, pulse 100.

Passed a restless night last night.

9*th*. 6 A. M., temperature 98⅖°, pulse 90. 6 P. M., temperature 99½°, pulse 100.

Has been quite sick to-day. Suffered from severe pain in the locality of the pedicle, which continued for two or three hours, and was followed by nausea and vomiting. Glass drainage-tube was removed, and a small rubber tube introduced.

10*th*. 8 A. M., temperature 98⅗°, pulse 90. 6 P. M., temperature 99°, pulse 92.

11*th*. 8 A. M., temperature 99½°, pulse 90.

15*th*. 8 A. M., temperature 98⅘°, pulse 76.

20*th*. 8 A. M., temperature 99⅘°, pulse 100.

*24th.* 8 A.M., temperature 99°, pulse 100.

*25th.* 8 A.M., temperature $98\frac{1}{2}$°, pulse 100.

Was taken with pain in the region of the pedicle at 10 A. M., on 24th, and vomited at intervals of two or three hours until this morning. She is now quite comfortable.

*30th.* 8 A. M., temperature $98\frac{1}{2}$°, pulse 76. From this time she made a perfect recovery.

